# An Immune-Related Signature Predicted Survival in Patients With Kidney Papillary Cell Carcinoma

**DOI:** 10.3389/fonc.2021.670047

**Published:** 2021-06-07

**Authors:** Junwen Shen, Rongjiang Wang, Yu Chen, Zhihai Fang, Jianer Tang, Jianxiang Yao, Yuhang Ling, Lisha Zhang, Xu Zhang

**Affiliations:** ^1^ The First Hospital of Huzhou, Huzhou, China; ^2^ Zhejiang University of Science and Technology, Hangzhou, China

**Keywords:** kidney papillary cell carcinoma, immune-related, genes, prognostic, signature

## Abstract

Immune-related genes are important factors in tumor progression. The main aim of this study was to identify the immune-related genes in kidney papillary cell carcinoma (pRCC) patients. We downloaded RNAseq data and clinical information of pRCC patients from the TCGA database and retrieved the immune-related genes list from Immport. From the data, we mined out 2,468 differential expression genes (DEGs) and 183 immune-related DEGs. Four hub DEGs (*NTS*, *BIRC5*, *ELN*, and *CHGA*) were identified after conducting Cox analysis and LASSO analysis. Moreover, the prognostic value of the signature based on four hub DEGs was verified using Kaplan–Meier analysis (P = 0.0041 in the training set and p = 0.021 in the test set) and ROC analysis (AUC: 0.957 in 1 year, 0.965 in 2 years, and 0.901 in 3 years in the training set, and 0.963 in 1 year, 0.898 in 2 years, and 0.742 in 3 years in the test set). Furthermore, we found that the high-risk score group had a higher percentage of B cells in the immune component, a higher expression of immune-related genes (*CTLA4*, *LAG3*, *PDCD1LG2*, and *TIGIT*), and a better immunotherapy response.

## Introduction

Kidney papillary cell carcinoma (pRCC) is the second most common type of renal cancer after renal clear cell carcinoma (1). It is worth noting that the first choice treatment method for pRCC patients is maximum resection of the tumor. However patients, who had done the resection of the tumor, face the challenge of disease progression (2). Therefore, immunotherapy has become the latest choice for advanced metastatic pRCC patients (3). Nivolumab, a programmed death 1 (PD-1) immune checkpoint inhibitor monoclonal antibody, was approved as monotherapy in 2015 for metastatic RCC patients after treatment with a VEGF-targeting agent. In April 2018, the combination of nivolumab and ipilimumab, a CTLA-4 inhibitor, was approved for intermediate- and poor-risk, previously untreated patients with metastatic RCC. Then, in 2019, combination therapies consisting of pembrolizumab (anti-PD-1) or avelumab [anti-PD-ligand (L) 1] with axitinib (a VEGF receptor tyrosine kinase inhibitor) were also approved to treat metastatic RCC and were likely to produce dramatic shifts in the therapeutic landscape (4, 5). According to EAU guidelines (6), immunotherapy was a second-line therapy option for advanced metastatic pRCC patients by the end of 2020.

The prognostic value of immune-related genes has become a subject of persistent focus in cancer research. Some special immune-related genes or the signature has a significant survival prognostic value for tumor patients (7, 8). However, the studies which focused on the relationship with immune related genes and pRCC were few. The main aim of this study was to identify the immune-related genes in pRCC patients.

## Methods and Results

### Identification of Immune-Related Genes Using Differential Expression Data

We downloaded the gene expression RNAseq data and clinical phenotype of TCGA kidney papillary cell carcinoma (KIRP) from the UCSC Xena database (https://xenabrowser.net/datapages/). The data was then pre-processed with the cancer tissue using the following steps: 1. Exclusion of the samples without clinical data; 2. Exclusion of genes with FPKM <1 from all samples. We used the R package “limma” (condition: adjusted P Value <0.01, and |logFC|>2) to find the differentially expressed genes. Furthermore, we downloaded the list of the immune-related genes from the ImmPort Portal database (https://www.immport.org/home/) which contained 2,483 immune-related genes (9).

**Figure 1:**
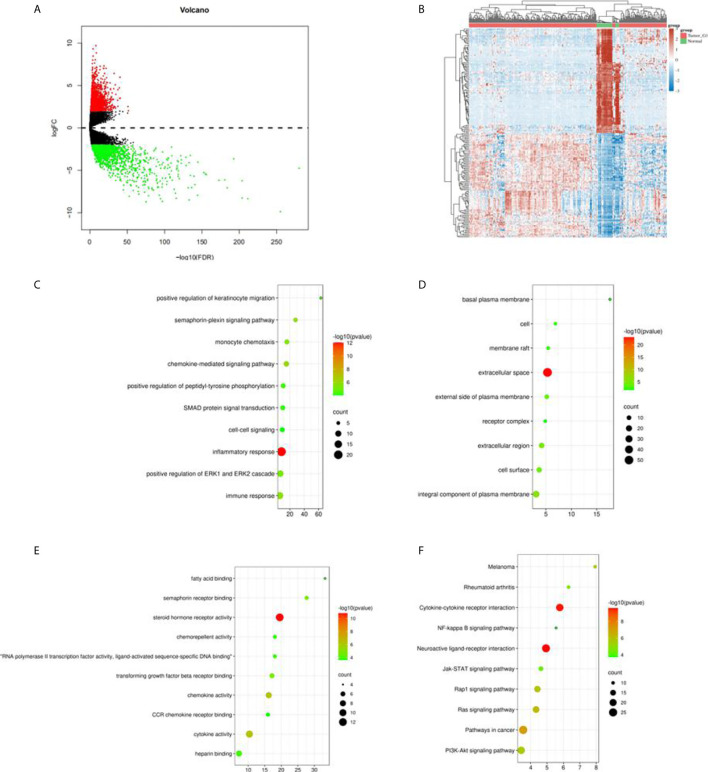
Differential expression genes in TCGA KIRC data and enrichment analysis. **(A)** Volcano plot in the differential expression genes (DEGs) in TCGA KIRC data; **(B)** the heatmap of top 50 DEGs; **(C)** the biological process in GO term; **(D)** the cellular component in GO term; **(E)** the molecular function in GO term; **(F)** the pathway analysis in KEGG function.

In total, we obtained 321 sample data which contained 289 cases of kidney papillary cell carcinoma (pRCC) and 32 normal samples from the TCGA KIPR data. After the data was pre-processed, all the 289 pRCC and 32 normal samples were enrolled. Among them, 2,468 differentially expressed genes (DEGs): 638 up-regulated genes and 1,830 downregulated genes (**Figures 1A, B**) were identified using R package “limma” (10). Intersection of the immune-related genes and 2,483 DEGs resulted in the identification of 183 immune-related DEGs.

### Functional and Pathway Enrichment Analysis of Immune-Related DEGs

We used an online analysis tool created by David (https://david.ncifcrf.gov/) to perform gene ontology (GO) analysis and Kyoto Encyclopedia of Genes and Genomes (KEGG) analysis for all the 183 immune-related DEGs (11).

GO analysis classified the DEGs into three groups: molecular function group, biological process group, and cellular component group. The biological results revealed that DEGs were primarily enriched in inflammatory response, positive regulation of ERP1 and ERK2 cascade, and immune response (**Figure 1C**). The cellular component results indicated that DEGs were mainly enriched in extracellular space, an integral component of the plasma membrane (**Figure 1D**). The molecular function results showed that DEGs were mainly enriched in semaphorin receptor binding, chemokine activity, and cytokine activity (**Figure 1E**). Moreover, KEGG analysis indicated that the DEGs’ pathways were mainly enriched in neuroactive ligand–receptor interaction and cytokine–cytokine receptor interaction (**Figure 1F**).

### Training and Test Sets

We divided 289 samples into a training set (n = 203) and a test set (n = 86). The two sets satisfied the following criteria: 1. Samples were randomly divided into two sets; 2. Age, clinical stage, and follow-up time between the two sets were similar. The clinical information obtained from the two sets is shown in **Table 1**.

**Table 1:** The clinical information of the training set and the test set.

	Training set	Test set
Cases (n)	203	86
Age (>60 years old; <60 years old, n)	115, 88	55, 31
Sex (male, female, n)	154, 49	58, 28
T stage (T1, T2, T3, T4, Tx, n)	134, 26, 40, 2, 1	60, 6, 19, 0, 1
N stage (N0, N1, N2, Nx, n)	35, 17, 1, 150	15, 7, 3, 61
M stage (M0, M1, Mx, n)	72, 6, 125	30, 3, 53
Grade stage (I, II, III, IV, n)	122, 18, 53, 10	58, 3, 19, 6
Statue (alive, dead, n)	175, 28	70, 16

### Identification of Survival Genes From the Immune-Related DEGs in the Training Set

Firstly, we used univariate Cox proportional hazard regression to analyze the RNAseq expression and survival date of all the 183 DEGs in the training set. Secondly, LASSO Cox regression analysis was used to analyze the valuable DEGs. Finally, multivariate Cox regression analysis was performed to identify survival DEGs. R package “survival” and “glmnt’ were used for the above calculation, and p <0.05 was considered to be statistically significant.

**Figure 2:**
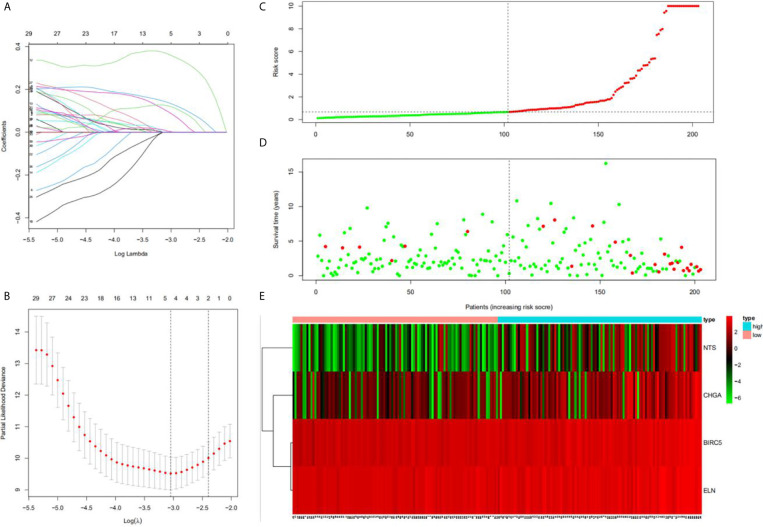
Construction of the hub DEGs and risk score prognostic value. **(A, B)** The determination of the number of factors by the LASSO analysis; **(C)** the distribution of risk score patients; **(D)** the survival state of patients; **(E)** a heatmap of hub DEGs in the training set.

Univariate Cox regression analysis results identified 39 DEGs (**Table 2**), of which five were left after conducting LASSO Cox regression analysis for a thousand times (**Figures 2A, B**). Finally, four DEGs (*NTS*, *BIRC5*, *ELN*, and *CHGA*) were selected as the survival genes after conducting multivariate Cox regression analysis (**Table 3**). The survival state, risk score, and heatmap of four hub genes in the training set were shown (**Figures 2C–E**).

**Table 2:** Top 10 genes in univariate Cox analysis.

Gene id	HR	HR.95L	HR.95H	P value
BIRC5	2.155273	1.648415	2.817979	1.98E-08
CHGA	1.268253	1.150524	1.398028	1.75E-06
ELN	1.532013	1.256153	1.868453	2.54E-05
CCL19	1.297936	1.13736	1.481184	0.000109
LEFTY2	1.329668	1.127774	1.567704	0.000696
IL17B	1.631201	1.226547	2.169357	0.000769
NR3C2	0.494363	0.327281	0.746742	0.000815
NTS	1.325878	1.122007	1.566794	0.000928
VTN	1.240989	1.077436	1.42937	0.00275
CR2	1.266519	1.076726	1.489766	0.004339

**Table 3:** The four hub genes in multivariate Cox analysis.

Gene id	coef	HR	HR.95L	HR.95H	P value
NTS	0.250656	1.284869	1.048373	1.574714	0.01573
BIRC5	0.465259	1.592426	1.189835	2.131239	0.001755
ELN	0.251223	1.285597	0.999556	1.653493	0.050406
CHGA	0.241936	1.273712	1.12858	1.437508	8.87E-05

### Analysis of Hub DEGs

**Figure 3:**
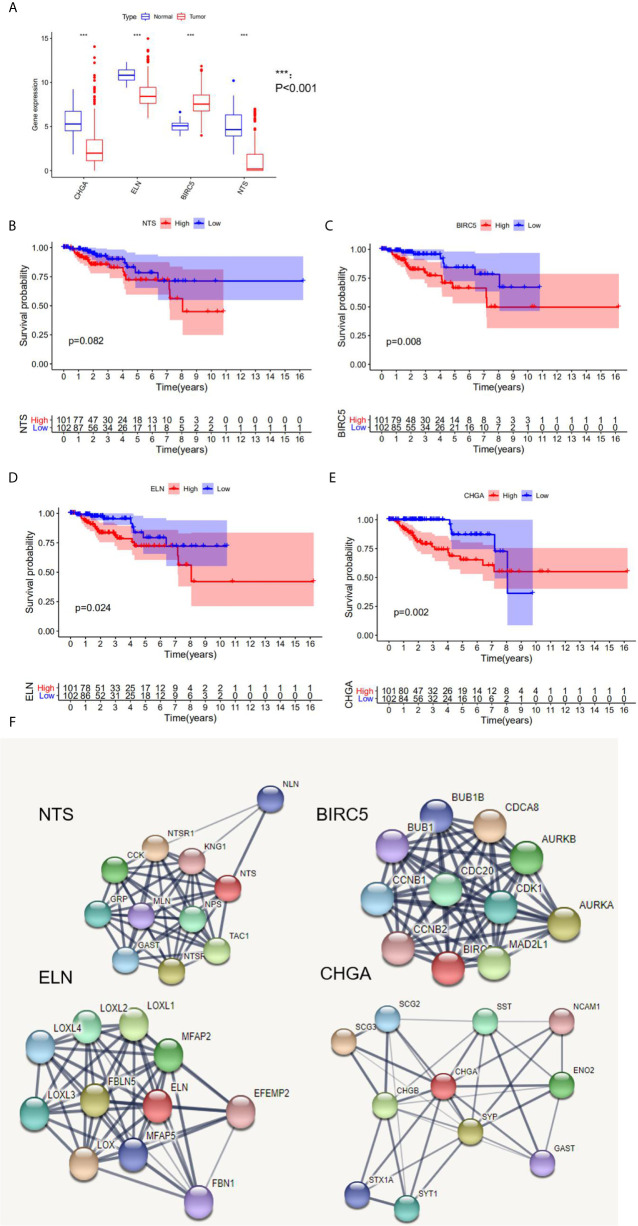
The analysis of hub DEGs’ expression, survival results, and protein–protein interaction. **(A)** The mRNA expression of four hub DEGs in normal *vs* tumor tissue; **(B–E)** the survival results in Kaplan-Meier analysis; **(F)** the protein-protein interaction of four hub DEGs.

**Figure 4:**
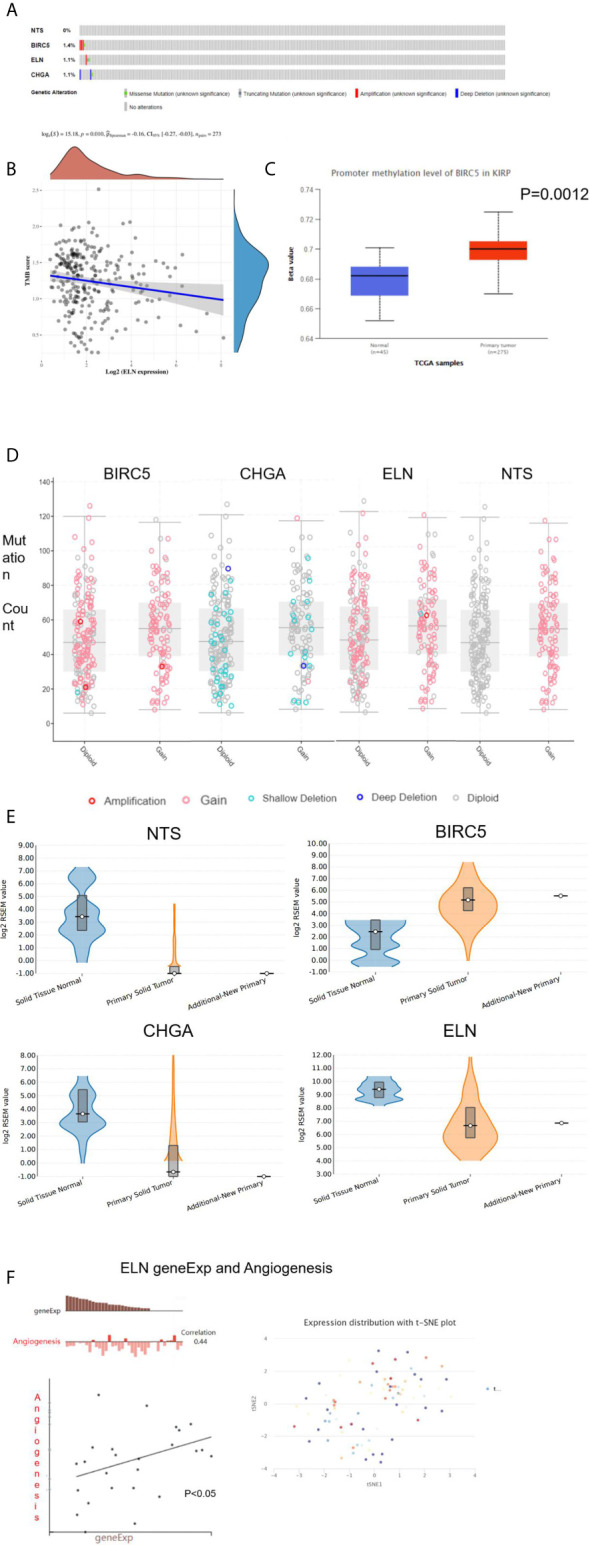
The analysis of hub DEGs’ DNA methylation, TMB, CBV, and variable shear. **(A)** The genetic alterations of four hub DEGs; **(B)** The correlation between ELN geneEpx and TMB; **(C)** the correlation between BIRC5 geneEpx and DNA methylation level; **(D)** the correlation between hub DEGs geneEpx and CNV; **(E)** the correlation between hub DEGs geneEpx and variable shear; **(F)** the correlation between ELN geneEpx and angiogenesis.

Three hub genes (*NTS*, *ELN*, and *CHGA*) had lower mRNA expression, and one hub gene (*BIRC5*) had higher mRNA expression in primary cancer tissue compared to the normal kidney tissue (**Figure 3A**). In addition, Kaplan–Meier analysis results indicated that three genes (*BIRC5*, *ELN*, and *CHGA*) were survival-related (**Figures 3B–E**). We used the STRING online tool (https://string-db.org/) (12) to determine the protein–protein interactive relationship of the four hub genes (**Figure 3F**). The DNA methylation analysis function of UALCAN (http://ualcan.path.uab.edu/) (13) revealed that DNA hyper-methylation occurred as a result of high-level mRNA expression of *BIRC5* (p = 0.0013, **Figure 4C**).

The cbioportal website (https://www.cbioportal.org/) (14) was then used to explore the genetic alterations (**Figure 4A**) and copy numbers (CNV, **Figure 4D**) of the four hub genes. On the other hand, the ACB website (https://www.aclbi.com/static/index.html) (15) indicated that *ELN* gene expression was correlated with tumor mutation load (TMB, p = 0.01, **Figure 4B**). We also investigated the variable shear situation of the four hub genes in the TSVdb website (http://www.tsvdb.com/) (16). Results obtained from the online single-cell library of CancerSea (http://biocc.hrbmu.edu.cn/CancerSEA/, **Figure 4E**) **(** 17) indicated that the mRNA expression of *ELN* was associated with angiogenesis (correlation = 0.44, p < 0.05, **Figure 4F**).

### Prognostic Value Verification of the Four-mRNA Signature in the Training Set and Test Set

The risk score of each patient was calculated based on the coefficients: Risk score = (0.250656 * Exp NTS) + (0.465259 * Exp BIRC5) + (0.251223 * Exp ELN) + (0.241936 * Exp CHGA). We found that the four-mRNA signature risk score was an independent factor in multivariate Cox analysis (**Table 4**).

**Table 4:** The clinical classifier and risk score in multivariate Cox analysis.

	coef	HR	HR.95L	HR.95H	P value
age	0.005597	1.005613	0.976774	1.035303	0.706164
gender	0.465879	1.593414	0.707722	3.587523	0.26055
grade	0.470943	1.601504	1.019282	2.516297	0.041072
T	0.10912	1.115296	0.757509	1.642073	0.580355
M	−0.00442	0.995594	0.723228	1.370533	0.9784
N	0.082667	1.08618	0.664299	1.775988	0.74176
Risk Score	0.048964	1.050183	1.028824	1.071985	3.00E-06

A total of 203 samples in the training set were divided into high-risk score group (n = 101) and low-risk score group (n = 102) according to the risk score. A comparison of the two groups indicated that the high-risk score group had a higher mortality rate, while the low-risk score group had a large number of surviving patients (**Figures 3C–E**). Moreover, similar results were observed when 86 samples in the test set were divided into high-risk score group (n = 42) and low-risk score group (n = 44).

**Figure 5:**
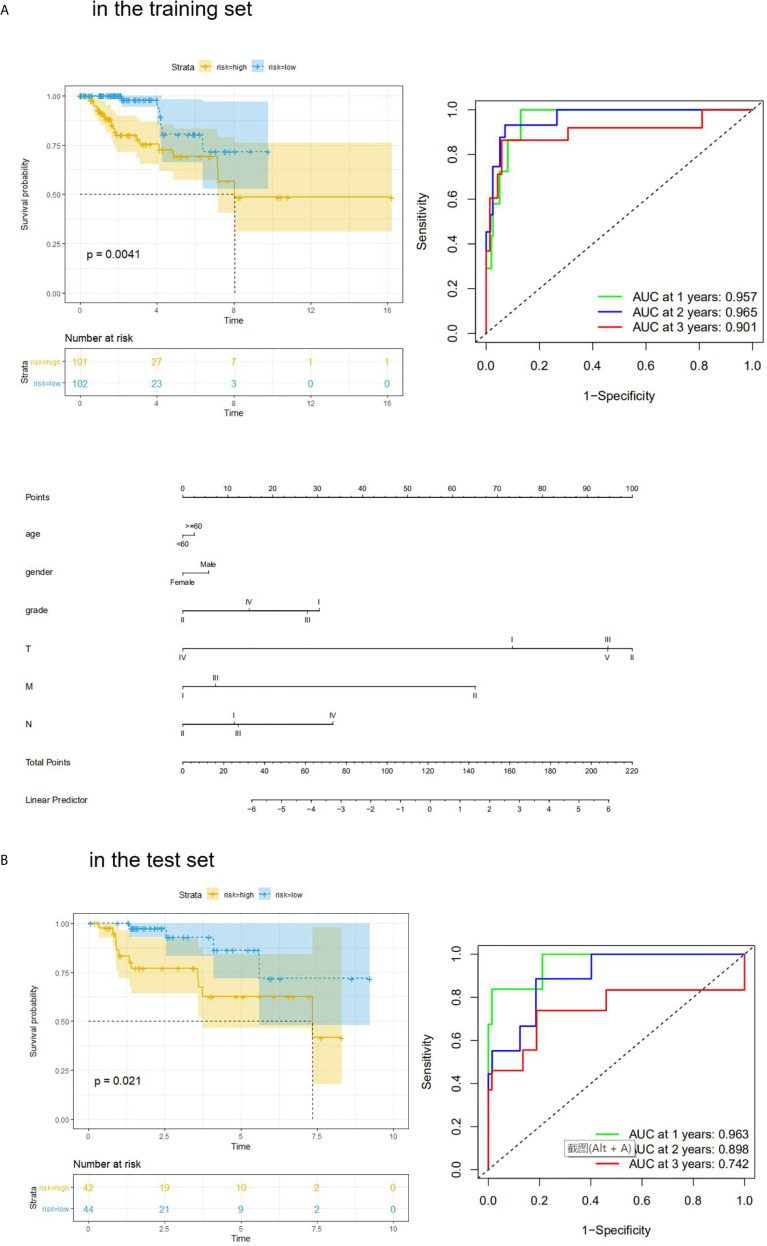
KM analysis and ROC analysis of risk score value in both sets. **(A)** KM analysis, ROC analysis, and clinical nomogram model of risk score value in the training set; **(B)** KM analysis, and ROC analysis of risk score value in the test set.

We also performed Kaplan**–**Meier analysis and ROC analysis for risk score on the training set and test set. Kaplan**–**Meier analysis in the training set found that the high-risk score group had a significantly shorter overall survival time (p = 0.0041, **Figure 5A**), while the ROC curves showed that the four-mRNA signature had good accuracy with 0.957 in 1 year, 0.965 in 2 years, and 0.901 in 3 years (**Figure 5A**). On the other hand, Kaplan**–**Meier analysis in the test set indicated that the high score group also had a significantly shorter overall survival time (p = 0.021, **Figure 5B**), and the time-dependent ROC curves had good accuracy (0.963 in 1 year, 0.898 in 2 years, and 0.742 in 3 years, **Figure 5B**). Furthermore, nomogram models for the training set were drawn (**Figure 5B**).

### Correlation Between Risk Score and Clinical Feature

**Figure 6:**
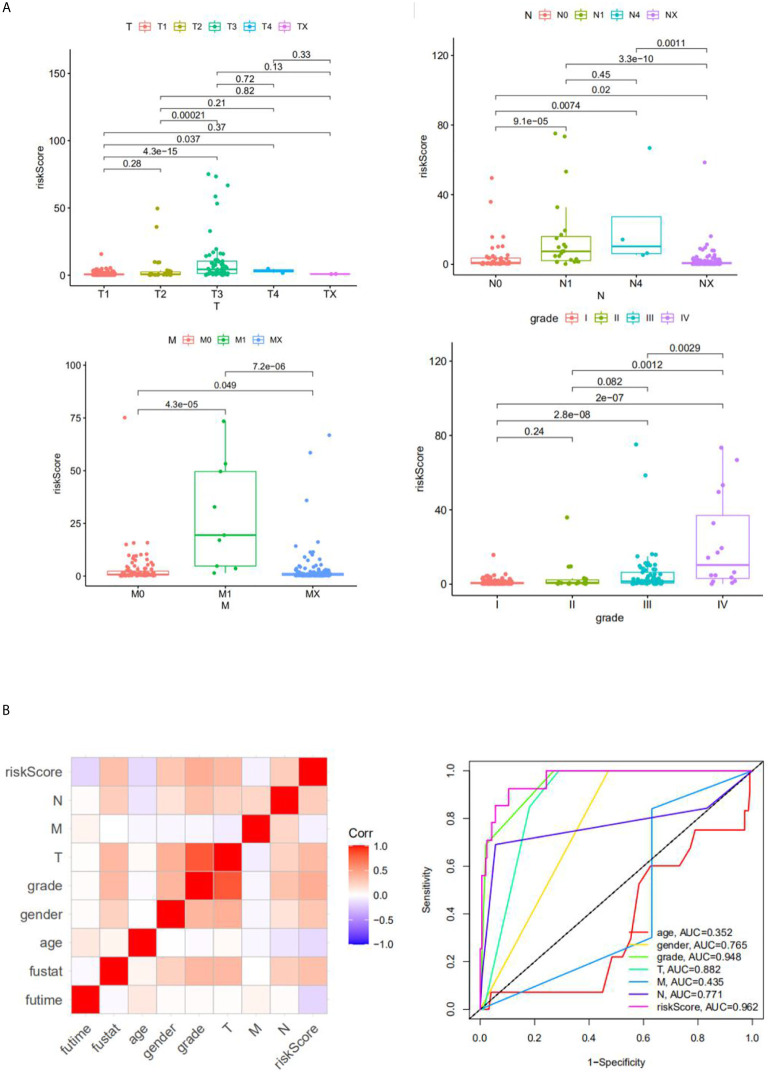
The correlation between the risk score and clinical classifier. **(A)** The correlation between the risk score and TNM grade information; **(B)** the ROC.

We analyzed the correlation between risk score and different clinical information (tumor, lymph node, metastasis degrees, and grades). The results showed that the risk score had significant differences in tumor, lymph node, metastasis degrees, and grades (**Figure 6A**). Moreover, time-dependent ROC analysis found that the four-mRNA signature had better accuracy compared to other clinical features (**Figure 6B**).

### The Relationship Between the Risk Score and Immune Cell Component, Expression of Immune-Related Genes, and Immunotherapy Response

**Figure 7:**
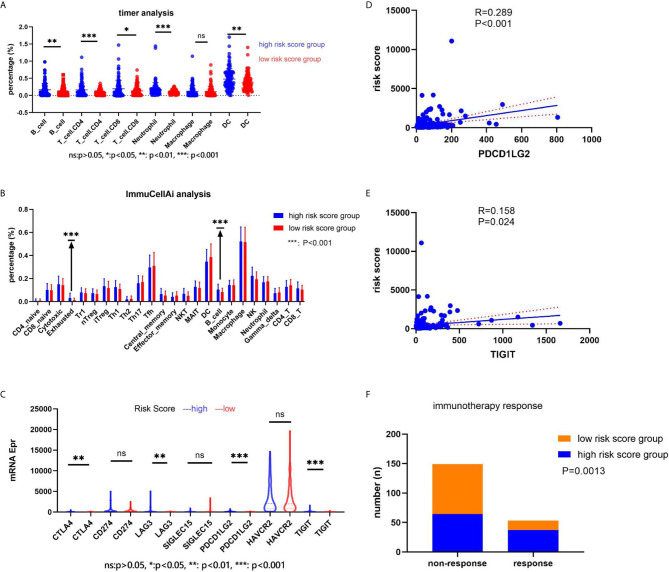
The relationship between the risk score and immune cell component, the immune-related genes’ expression, and the immunotherapy response. **(A)** The immune cell component in the web of timer; **(B)** the immune cell component in the web of ImmuCellAI; **(C)** the immune-related genes’ expression in two groups; **(D, E)** the correlation between the risk score and the immune-related genes; **(F)** the immunotherapy response.

We explored the immune cell component using two online analysis tools (TIMER: https://cistrome.shinyapps.io/timer/ (18) and ImmuCellAI: http://bioinfo.life.hust.edu.cn/ImmuCellAI/ (19)). TIMER analysis results showed that a significantly higher percentage of B cells (P = 0.0029), T cell + CD4 (P = 0.0002), T cell + CD8 (P = 0.0029), Neutrophil cells (P = 0.0004), and DC cells (P = 0.0047) would appear in the high-risk score group in the training set (**Figure 7A**). On the other hand, ImmuCellAI analysis results indicated that the high-risk score group had a higher percentage of Exhausted cells (P = 0.00003) and B cells (P = 0.0003) (**Figure 7B**). We also analyzed the expression of seven immune-related genes (*CTLA4*, *CD274*, *LAG3*, *SIGLEC15*, *PDCD1LG2*, *HAVCR2*, and *TIGIT*) and correlated the expression with the risk score. The results showed that *CTLA4*, *LAG3*, *PDCD1LG2*, and *TIGIT* had a higher expression in the high-risk score group (P < 0.05, **Figure 7C**). In addition, the expression of *PDCD1LG2* and *TIGIT* genes correlated with the risk score value (P < 0.05, **Figures 7D, E**). According to the immunotherapy response results obtained after ImmuCellAI analysis, the high-risk score group had a better immunotherapy response (P = 0.0013, **Figure 7F**).

## Discussion

In this study, we identified four hub mRNA genes (*NTS*, *BIRC5*, *ELN*, and *CHGA*) using univariate and multivariate Cox analysis and LASSO analysis. Pro Qiu team have reported that *NTS* was a neurotensin receptor that participates in the colorectal cancer tissue (20). The pro AKter team found that *NTS* had the function of cell migration and invasion in gastro-intestinal and cardiovascular functions (21). The Ye team also found that the *NTS* gene activates the Wnt/*β*-catenin signaling pathway, thereby promoting tumor metastasis (22). *BIRC5* is a member of the apoptosis inhibitor gene family, which encodes regulatory proteins that prevent apoptotic cell death (23). It regulates several types of cancer cells by activating a multiple-step cell apoptosis process (24, 25). *ELN* encodes the elastin protein, which is a key protein in the tumor microenvironment (26). Moreover, the protein encoded by the *CHGA* gene is a member of the chromogranin/secretogranin family of neuroendocrine secretory proteins (27). Its gene product is a precursor of the peptides which act as autocrine or paracrine negative modulators of the neuroendocrine system (28).

We then verified the prognostic value of the signature in both training and test sets. Kaplan–Meier analysis showed that the high-risk score group had a bad survival time, while ROC analysis found that the AUC of the signature was excellent (0.957 in 1 year, 0.965 in 2 years, and 0.901 in 3 years in the training set). A previous study had developed a five-mRNA gene signature for pRCC patients and proved that the AUC of the signature was 0.82 (29). Furthermore, we conducted a correlation between the risk score and clinical classification and found that the risk score was correlated with the TNM stage. The above results convinced us that the signature had an accurate prognostic value.

Finally, we conducted an analysis of the immune component and found different immune components in the two risk score groups. TIMER and ImmuCellAI analyses results indicated that the high-risk score group had a higher percentage of B cells in the immune component. Moreover, we conducted a correlation between the risk score and expression of immune-related genes. Our results indicated that the high-risk score group had a higher expression level of *CTLA4*, *LAG3*, *PDCD1LG2*, and *TIGIT*. In addition, the high-risk score group had a better immunotherapy response.

In summary, this study has identified four hub immune-related genes (*NTS*, *BIRC5*, *ELN*, and *CHGA*) in pRCC patients. We also developed a signature of four hub genes which can act as an independent prognostic factor for overall survival. Our results suggest that pRCC patients with a high-risk score have a shorter survival time and a better immunotherapy response.

## Data Availability Statement

The original contributions presented in the study are included in the article/supplementary material. Further inquiries can be directed to the corresponding author.

## Ethics Statement

Ethical review and approval was not required for the study on human participants in accordance with the local legislation and institutional requirements. Written informed consent for participation was not required for this study in accordance with the national legislation and the institutional requirements.

## Author Contributions

JS wrote the paper. RW and LZ edited the paper. YC, ZF, JY, XZ, and YL analyzed the data. JT made the images out. All authors contributed to the article and approved the submitted version.

## Funding

The Zhejiang province medical and health project (2020KY937).

## Conflict of Interest

The authors declare that the research was conducted in the absence of any commercial or financial relationships that could be construed as a potential conflict of interest.
